# A prospective cohort study of low-back outcomes and alternative measures of cumulative external and internal vibration load on the lumbar spine of professional drivers

**DOI:** 10.5271/sjweh.3947

**Published:** 2021-04-27

**Authors:** Massimo Bovenzi, Marianne Schust

**Affiliations:** Clinical Unit of Occupational Medicine, Department of Medical Sciences, University of Trieste, Trieste, Italy; Unit Experimental Research on Occupational Health, Federal Institute for Occupational Safety and Health, Berlin, Germany

**Keywords:** acceleration magnitude, intraspinal force, low-back pain, vibration dose, whole body vibration

## Abstract

**Objective:**

The aim of this study was to compare the performance of alternative measures of cumulative lifetime vibration dose to predict the occurrence of low-back pain (LBP) outcomes in a cohort of 537 professional drivers investigated at baseline and over a two-year follow up period.

**Methods:**

The exposure data obtained in the EU VIBRISKS project were used to calculate alternative measures of either acceleration- (external) or force- (internal) based lifetime vibration doses. Vibration was measured in representative samples of machines and vehicles used by the drivers. Internal lumbar forces were calculated by means of anatomy-, posture-, and anthropometry-based finite element models. The relations of LBP outcomes to alternative measures of lifetime vibration doses were assessed by the generalized estimating equations method.

**Results:**

Metrics of cumulative vibration exposure constructed with either acceleration- or force-based methods were significantly associated with the occurrence of LBP outcomes. A measure of model fitting suggested that force-based doses were better predictors of LBP outcomes than acceleration-based doses. Models with force root-mean-square doses provided a better fit to LBP outcomes than those with force-peak doses.

**Conclusions:**

Measures of internal lumbar forces were better predictors of LBP outcomes than measures of external vibration acceleration although the exposure metrics constructed with the acceleration-based method have the advantage of greater simplicity compared to the force-based method. The differences between the models with force-based doses suggest that the cumulative health effects on the lumbar spine might depend on the integrated resulting total force over the entire exposure time rather than primarily on the force peaks.

Long-term exposure to whole-body vibration (WBV) at the workplace has been associated with an excess risk of low-back pain (LBP) outcomes (1). In 2015, the sixth European Working Conditions Survey (EWCS) reported that about 19% of the workforce employed in the 28 EU Member States were exposed to mechanical vibration at least a quarter of the time or more (2), mainly in agriculture and industry (38%) and construction and transport (36%). Among the 43 850 employees interviewed, the most reported health problem was backache (44%) followed by muscular pains in the arms (42%).

In the 2010 EWCS (3), drivers and mobile-plant operators showed a significantly greater prevalence of (low) back pain (55.1%) when compared with the reference category of teaching professionals (35.3%), resulting in a prevalence ratio of 1.36 [95% confidence interval (CI) 1.18– 1.58] after adjustment for individual-level risk factors.

Low-back disorders among professional drivers are considered to be of multifactorial origin since, in addition to individual characteristics, driving tasks involve exposures to several occupational risk factors such as WBV, excessive postural demands, and unfavorable psychosocial load (4).

To implement measures protecting workers from the adverse health effects of WBV, the EU Directive 2002/44/EC on mechanical vibration (5) established daily exposure action values (EAV) and exposure limit values (ELV) for WBV generated by machinery at the workplace. The EAV and ELV are based on the measurement of vibration acceleration according to ISO standard 2631-1 (6), and the daily exposure to WBV is evaluated in terms of either equivalent acceleration over an 8-hour period according to a second power time-dependency (*A*(8) in ms^−2^ r.m.s.) or the vibration dose value according to a fourth power time-dependency (VDV in ms^−1.75^). *A*(8) and VDV are metrics of external vibration acceleration load and may be poorly correlated with the internal load acting on the lumbar spine (7). In particular, there is some evidence that the current metrics of WBV exposure might underestimate the severe health effects of vibration that contain shocks (8).

Recently, international standard ISO 2361-5 has developed a procedure to calculate the compressive forces generated by WBV containing multiple shocks (9). The internal lumbar forces are calculated by means of anatomy-based finite element (FE) models of the seated human and adapted to different sitting postures and individual anthropometric data of representative groups of European drivers (10). In our previous cohort study of professional drivers recruited in the Italian arm of the EU VIBRISKS research project, the *daily* acceleration-based EU metrics *A*(8) and VDV were poorer predictors of LBP outcomes than the *daily* force-based compressive stress on the lumbar spine calculated according to ISO 2631-5 (9, 11). However, it is unlikely that measures of daily vibration exposure are suitable for the assessment of the risk of long-term adverse health effects such as disorders of the lumbar spine.

The aim of the present study is twofold, namely, to: (i) investigate whether measures of *cumulative lifetime* force-based vibration exposure are associated with the occurrence of low back symptoms in the professional drivers of the VIBRISKS study; and (ii) compare the performance of *lifetime* force-based doses to predict LBP outcomes with that of *lifetime* acceleration-based doses calculated according to a second power time dependency method (6) as reported in our previous study of the VIBRISKS cohort (12).

## Methods

### Study population

The study cohort included 537 male professional drivers of earth-moving machines in marble quarries (N=124), fork-lift trucks, freight-container tractors or mobile cranes in marble laboratories, dockyards, or paper mills (N=169), and garbage trucks or buses in public utilities (N=244). They were investigated at baseline and then participated annually in one (N=220) or two (N=317) follow up surveys. A minimum of one year of professional driving in the current job was the basic requirement for inclusion of drivers in the study population. Written informed consent to the study was obtained from employers and employees at each company. Further details on the recruitment and the response rate of the driver groups have been reported in our previous epidemiological study of the same cohort (12).

### Questionnaire and low-back pain outcomes

Personal sociodemographic characteristics, occupational history in current and previous jobs, daily and cumulative duration of driving on specific machines or vehicles, physical demands while driving, and psychosocial work environment were investigated by means of a structured questionnaire developed within the VIBRISKS research project (13). The several sections of the questionnaire have been described in detail in previous papers (12, 14). Briefly, perceived physical demands at work were evaluated by means of a combined approach of direct observation of working conditions (photographs and video) and the subject’s self-assessment during the interview. A measure of perceived physical work demands was constructed and categorized into four grades of increasing physical load (mild, moderate, hard, very hard) by rating the frequency of manual activities (eg, lifting loads) and the duration of awkward postures while driving (14). A measure of the perceived psychosocial work environment was derived from questions concerning job decision, job support, and job satisfaction (15) and categorized into four grades of increasing psychosocial load (good, reasonable, a little poor, poor). More details on the response scales used to grade the measures of physical and psychosocial load at work have been reported in a previous paper (12).

Table 1 reports the characteristics of the study population at baseline.

**Table 1:** Characteristics of the professional drivers (N=537) at the cross-sectional study.

Characteristics	N (%)	Median (quartiles)
Driving occupation		
Industry	293 (54.6)	
Public utilities	244 (45.4)	
Age (years)		41 (35–47)
BMI (kg^2^)		25.8 (23.9–28.4)
Smoking		
Never	246 (45.8)	
Ex-smokers	112 (20.9)	
Current smokers	179 (33.3)	
Drinking	345 (64.3)	
Married	387 (72.1)	
Education (years)		
≤6	35 (6.5)	
7–12	355 (66.1)	
>12	147 (27.4)	
Physical activity		
Never	237 (44.1)	
<1 per week	64 (11.9)	
1–3 per week	210 (39.1)	
Every day	26 (4.8)	
Car driving (km/year)		
<8000	145 (27.0)	
8000–24 000	347 (64.6)	
>24 000	45 (8.4)	
Seniority in current job (years)		12 (5–20)
Seniority in driving occupations (years)		16 (8–23)
Physical workload		
Mild	185 (34.5)	
Moderate	144 (26.8)	
Hard	106 (19.7)	
Very hard	102 (19.0)	
Psychosocial work environment		
Good	151 (28.1)	
Reasonable	134 (24.9)	
A little poor	164 (30.5)	
Poor	88 (16.4)	

Low-back symptoms occurred in the previous 12 months were defined as follows:

- LBP: pain or discomfort in the low-back area between the 12^th^ ribs and the gluteal folds (indicated in a figure), lasting ≥7 days but <30 days in the previous 12 months;
- Chronic LBP: daily experience of LBP or several episodes lasting >30 days in the previous 12 months;
- Sciatic pain: radiating pain in one or both legs (below the knee) in the previous 12 months.

### Vibration exposure, intraspinal forces, and measures of lifetime vibration dose

Vibration was measured on representative, randomly selected, samples of industrial machines and vehicles used by the professional drivers according to the recommendations of the International Standard ISO 2631-1 (6) and the VIBRISKS protocol (13).

Vibration was measured at the driver-seat interface with a semi-rigid mounting disc containing three uni-axial accelerometers. From one-third band frequency spectra (1–80 Hz) recorded from *x-*, *y-*, and *z-*directions, frequency-weighted accelerations (*a_wx_, a_wy_, a_wz_*) were obtained by using the weighting factors suggested in ISO 2631-1 (6). Daily vibration exposure was expressed in terms of 8-hour energy-equivalent frequency-weighted r.m.s. acceleration [*A*(8)] according to the EU Directive on mechanical vibration (5).

The intra-spinal forces were predicted using anatomy-based FE models. Representative tri-axial acceleration time histories from various machine types and working tasks were selected as an input to FE models. The measuring time varied from 300–1100 seconds. Ultimately, 19-time histories from as many machines/vehicles were available as inputs into the FE models. Some subjects operated a combination of two or three vehicles per working day, other subjects operated one machine exclusively. All of the time histories contained shocks in at least one direction according to several criteria for detecting shock (7). Impacts due to sitting down or losing the contact to the seat were eliminated. The median value of unweighted accelerations (*a_unw,z_* r.m.s.) the subjects were exposed to was 0.64 (range 0.29 – 1.04) ms^-2^ r.m.s.. The internal forces were predicted on the basis of transfer functions between unweighted vibration accelerations and vertebral forces. The transfer functions are derived from the results of validated anatomy-based FE models and depend on the posture, body mass (BM), body mass index (BMI) of the exposed subjects, as well as the magnitude of the measured acceleration (9). On the basis of 4 observed sitting postures of the drivers (photographs and video), 10 BM/BMI categories, and 2 ranges of acceleration magnitude (*a_unw,z_* < 0.65 ms^-2^, 0.65 ms^-2^ ≤ *a_unw,z_* ≤ 1.35 ms^-2^), 80 modifications of the FE model were created. These 80 model modifications delivered 17280 acceleration to spine-force transfer functions in total as a basis for calculating the force time histories (80 modifications of the model, 4 acceleration inputs (buttock, back, hands, feet), 3 acceleration directions, 6 spine levels, 3 spinal force directions). A description of the 4 observed sitting postures and the 10 BM/BMI categories, and the sophisticated procedure of data processing for the estimation of static and dynamic spinal forces based on FE models have been reported elsewhere (7).

In this study, several measures of cumulative lifetime vibration doses were calculated by combining an exposure quantity with a duration of vibration (table 2). The exposure quantities for acceleration-based doses are given by either the frequency-weighted r.m.s. acceleration of the machine vibration in the vertical direction (*a*_wz_) or the derived *A*(8)_z_ metric calculated according to the principle of “equal energy”. The choice of the *z*-axis (vertical) weighted acceleration for the calculation of acceleration-based doses was for purposes of comparison with the ISO 2361-5 method for the evaluation of exposure to multiple mechanical shocks, which is validated for exposures to peak acceleration signals up to 9.81 ms^-2^ in the *z*-direction (9). It should be noted that in this study the exposure quantities for acceleration-based doses (*a*_wz_, *A*(8)_z_) were derived from the time histories of vibration acceleration used for the calculation of force-based doses; as a result, they may differ from the values of acceleration measures reported in previous studies of the VIBRISKS cohort (12).

**Table 2:** Alternative measures of cumulative lifetime vibration dose. [t_d_ = daily exposure time (s); t_dj_ = daily exposure time (s) to condition (machine) j; t_mj_ =measurement time (s) of condition (machine) j over which S_j_ has been calculated; total driving days= (driving days/year)×(driving years) assuming identical exposure days per year; a_wz_ = frequency-weighted r.m.s. acceleration magnitude (z-direction); A(8)_z_ = 8-hour energy- equivalent r.m.s. acceleration magnitude (z-direction), calculated as [a_wz_ (t_d_/28800)^½^]; F_total,rms_ = r.m.s. value of force calculated from the internal forces in three directions (Euclidean vector); F_d,total,peak_ = daily dose of peak-to-peak value of force calculated from the internal forces in three directions (Euclidean vector); S_j_ = dynamic compressive stress of the lumbar spine due to vibration exposure to condition (machine) j defined as the sum of peak compressive forces acting on the area of a vertebra endplate (cm^2^); S_ed_ = daily compressive dose (ISO 2361-5).

Measures	Formula	Units
∑[*a_wz_*^2^*t*]	∑[*a_wz_*^2 ^× (t_d_ × total driving days)]	m^2^s^-3^
∑[*A*(8)*_z_*^2^*t*]	∑[*A*(8)*_z_*^2^× total driving days]	m^2^s^-4^
∑[F*_total,rms_*^2^*t*]	∑[force*_total,rms_*^2^× (t_d_ × total driving days)]	N^2^s
∑[F*_total,rms_t*]	∑[force*_total,rms_* × (t_d_ × total driving days)]	Ns
∑[F*_d,total,peak_* *t*^1/6^]	∑[daily force*_total,peak_* exposure × (total driving days)^1/6^]	N
	$F_{d,\mathrm{total},\mathrm{peak}}={\left(\sum_{j}F_{\mathrm{total},\mathrm{peak},j}^{6}\times \frac{t_{\mathrm{dj}}}{t_{\mathrm{mj}}}\right)}^{1/6}$	
∑[Sed*_peak_* *t*^1/6^]	∑[daily compressive dose*_peak_* × (total driving days)^1/6^]	MPa
	$S_{\mathrm{ed}}={\left(\sum_{j}S_{j}^{6}\times \frac{t_{\mathrm{dj}}}{t_{\mathrm{mj}}}\right)}^{1/6}$	

The exposure quantities for force-based doses are given by the r.m.s. value of force (F*_total,rms_*) and the daily dose of peak-to-peak values of force (F*_d,total,peak_*) calculated from the internal spinal forces. The forces in the three directions (anterior-posterior F_ap_, lateral left-right (symmetric) F_lat_, compressive-decompressive F_cd_) were aggregated in F*_total,rms_* and F*_d,total,peak_* by calculating the Euclidean vector (index ‘total’).

The daily compressive dose S_ed_ recommended by ISO 2361-5 (9) was also calculated (table 2). The metric S_ed_ (MPa) is a measure of whole-body multiple-shock exposure based on the estimation of vibration-induced dynamic compressive stress on the lumbar spine. S_ed_ is calculated from the sum of the peak compressive vertical forces depending on sitting posture, body mass and body mass index and acting on the area of a vertebra endplate over the daily exposure time. Peak is defined here as a maximum value of the additional compressive force between two consecutive mean value crossings in the force time histories. The size of the endplates at the six lumbar spine levels from T12/L1 to L5/S1 is derived from Seidel et al. (16). The 6^th^ power method to calculate S_ed_ is based on the Palmgren-Miner model with reference to fatigue fractures caused by repeated compressive loading of the human spine (9). F*_d,total,peak_* (N) is computed similar to S_ed_ with the 6^th^ power but using the Euclidean vector of peak-to-peak values instead of the compressive peaks and without relation to the endplate areas.

To determine exposure duration for each driver, questionnaire data, information obtained by interviewing employees and employers, and company records were used to estimate daily and weekly exposure to WBV expressed in driving hours, as well as the total duration of exposure to WBV in full-time driving years. In addition, samples of driving activities were monitored by a digital chronometer to estimate the actual duration of vibration exposure during a typical workday and over a one-week period (17). More details on the methods to estimate the duration of daily and lifetime vibration exposures are reported elsewhere (12).

The dynamic internal forces and the daily compressive dose S_ed_ (spinal stress values) were calculated for each of the six lumbar spine levels from T12/L1 to L5/S1. The values are different at each spine level. They depend on factors like eg, sitting posture, BM/BMI, and range of external acceleration magnitude as mentioned above. In this study, data analysis was carried out on the basis of the highest among the six spinal stress values.

Table 3 reports median values (quartiles) of the measures of the acceleration-based and force-based lifetime vibration doses at baseline in the professional drivers.

**Table 3:** Measures of acceleration-based and force-based vibration doses in the professional drivers at baseline, (see table 2 for the definitions of the alternative measures of vibration dose).

Measures of vibration dose	Median (quartiles)
Total driving time (hours)	12 600 (4320–26 400)
*a_wz_* (ms^-2^ r.m.s.)	0.46 (0.35–0.53)
*A*(8)*_z_* (ms^-2^ r.m.s.)	0.27 (0.25–0.33)
F*_total,rms_* (N)	28.8 (20.2–32.1)
F*_d,total,peak_* (N)	516 (398–666)
S_ed_ (MPa)	0.28 (0.21–0.38)
∑[*a_wz_*^2^*t*] (×10^7 ^m^2^s^-3^)	1.23 (0.50–2.55)
∑[*A*(8)*_z_*^2^*t*] (×10^3 ^m^2^s^-4^)	0.44 (0.18–0.87)
∑[F*_total,rms_*^2^*t*] (×10^11 ^N^2^s)	0.40 (0.15–0.96)
∑[F*_total,rms_t*] (×10^9 ^Ns)	1.69 (0.74–3.11)
∑[F*_d,total,peak_* *t*^1/6^] (×10^3 ^N)	1.92 (1.35–2.53)
∑[Sed*_peak_* *t*^1/6^] (MPa)	1.07 (0.74–1.43)

### Data analysis

Continuous variables were summarized with the median as a measure of central tendency and quartiles as measures of dispersion. Categorical data were tabulated into contingency tables. Point and period prevalence and cumulative incidence were calculated according to conventional epidemiological methods.

The associations between LBP (binary) outcomes and individual- and work-related explanatory variables were assessed by means of the generalized estimating equations (GEE) method to account for the within-subject dependency of the observations over time. Odds ratios (ORs) and robust 95% CI were estimated from the GEE logistic regression coefficients and their standard errors. To investigate the temporal sequence of the relationship between the occurrence of LBP outcomes and predictor variables, GEE-logistic regression analysis was performed with a time-lag model, in which the binary outcome variable for subject *i* at time-point *t* (Y*_it_*) was related to independent variable(s) *k* for subject *i* measured at time-point *t* – 1 (X*_ikt_*_– 1_), ie, at one time-point earlier.

Measures of either acceleration-based or force-based lifetime vibration doses entered the logistic model as time-dependent continuous or categorical variables, while other individual- or work-related covariates were included as time-dependent categorical variables, except for age which was treated as a time independent continuous variable (age-at-entry). Interactions between independent variables were assessed by adding appropriate product terms to the GEE logistic models. All models included a linear term for time effect. The Quasi-likelihood Information Criterion (QIC), a modification of the Akaike’s Information Criterion (AIC), was used to compare the fit of GEE longitudinal models including alternative measures of cumulative lifetime vibration doses (18). The models with the smallest QIC values were chosen as the best-fitting models for the relation between LBP outcomes and vibration doses. By analogy with the strength of evidence rules suggested for the AIC method (19), the following guidelines for selecting the best-fitting model were adopted: ΔQIC ≤2 suggests no difference in the fit between models; 4≤ ΔQIC ≤7 tends to give support for the model with the smaller QIC; ΔQIC >10 means that the model with the smaller QIC provides a substantially better fit to the data.

## Results

### Occurrence of low-back pain outcomes

At the cross-sectional survey, the prevalences of LBP, chronic LBP, and sciatic pain were 12.7, 7.5, and 23.1%, respectively. Over the follow-up time, there were 79 new cases of LBP, 46 new cases of chronic LBP and 90 new cases of sciatic pain, giving rise to cumulative incidences of 16.8, 9.3 and 21.8%, respectively. Since there were 83 individuals who recovered from low-back symptoms during the follow up, the overall period prevalence of any LBP outcomes in the professional drivers was about 67%.

### Relation of low-back pain outcomes to acceleration- and force-based vibration doses

The associations between LBP outcomes and the alternative measures of cumulative lifetime vibration dose treated as either continuous or categorical (quartiles) variables are reported in tables 4 and 5, respectively. Overall, after adjustment for potential confounders acceleration-based doses ([*a_wz_*^2^*t*], [*A*(8)*_z_*^2^*t*]), force r.m.s.-based doses ([F*_total,rms_*^2^*t*], [F*_total,rms_t*]), and force peak-based doses ([F*_d,total,peak_ t*^1/6^], [Sed*_peak__t_*^1/6^]) were significantly associated with the occurrence of LBP outcomes over time, although to a different extent. Nevertheless, the information criterion QIC for model fitting suggested that force-based doses were better predictors of LBP outcomes than acceleration-based doses, mainly when the measures of lifetime vibration doses were included as continuous variables in the GEE logistic models: compared with the acceleration model [*a_wz_*^2^*t*], calculated according to a second power time dependency, the force models showed smaller QIC values with ΔQIC of 7–12 units for LBP, 5–13 units for chronic LBP, and 9–17 units for sciatic pain. There were differences between force-based doses in the fit to LBP outcomes: test for trend and ΔQIC values revealed that force r.m.s.-based doses performed better for the prediction of LBP outcomes than force peak-based doses (eg, sciatic pain: ΔQIC of 5–8 units for models with continuous data, ΔQIC of 5–16 units for models with categorical data).

**Table 4:** Continuous relationships of 12-month low-back pain (LBP) outcomes to alternative measures of cumulative lifetime vibration dose among professional drivers (N=537, observations=854). Changes in odds ratio (OR) for a change in 10^n^ dose units are shown, (see table 2 for the definitions of the measures of cumulative lifetime vibration dose). [CI=confidence interval.]

Measures of vibration dose	LBP			Chronic LBP			Sciatic pain		
		
	OR (95% CI) ^a,b^	Wald test (P-value)	QIC (Δ) ^c^	OR (95% CI) ^a,b^	Wald test (P-value)	QIC (Δ) ^c^	OR (95% CI) ^a,b^	Wald test (P-value)	QIC (Δ) ^c^
∑[*a_wz_*^2^*t*] (10^7 ^m^2^s^-3^)	1.09 (0.98–1.22)	2.47 (0.12)	756 (0)	1.13 (0.99–1.29)	3.42 (0.064)	533 (0)	1.15 (1.02–1.29)	5.48 (0.019)	998 (0)
∑[*A*(8)*_z_*^2^*t*] (10^3 ^m^2^s^-4^)	1.51 (1.05–2.16)	4.91 (0.027)	753 (-3)	1.65 (1.08–2.51)	5.45 (0.020)	530 (-3)	1.69 (1.17–2.44)	7.75 (0.005)	994 (-4)
∑[F*_total,rms_*^2^*t*] (10^11 ^N^2^s)	1.77 (1.21–2.59)	8.68 (0.003)	744 (-12)	2.03 (1.35–3.07)	11.5 (0.0007)	521 (-12)	1.96 (1.41–2.72)	15.9 (0.0001)	981 (-17)
∑[F*_total,rms_t*] (10^9 ^Ns)	1.25 (1.07–1.45)	8.06 (0.0045)	744 (-12)	1.34 (1.12–1.60)	10.5 (0.0012)	520 (-13)	1.29 (1.13–1.47)	14.9 (0.0001)	982 (-16)
∑[F*_d,total,peak_* *t*^1/6^] (10^3 ^N)	1.53 (1.02–2.27)	4.31 (0.038)	749 (-7)	1.67 (1.01–2.76)	3.99 (0.046)	528 (-5)	1.65 (1.21–2.25)	9.92 (0.0016)	989 (-9)
∑[Sed*_peak_* *t*^1/6^] (MPa)	2.04 (1.06–3.95)	4.53 (0.033)	749 (-7)	2.54 (1.09–5.88)	4.71 (0.030)	527 (-6)	2.46 (1.46–4.17)	11.3 (0.0008)	987 (-11)

a ∑[*a_wz_*^2^*t*], ∑[*A*(8)*_z_*^2^*t*]: OR adjusted by age-at-entry, body mass index, physical work load, psychosocial work environment, and follow up time.

b ∑[F*_total,rms_*^2^*t*] to ∑[Sed*_peak_* *t*^1/6^]: OR adjusted by age-at-entry, physical work load, psychosocial work environment, and follow up time.

c QIC is the quasi-likelihood information criterion for the comparison between models. QIC difference (Δ) is calculated as the difference between the QIC value for a specific exposure model and the model including ∑[*a_wz_*^2^*t*] calculated according to a second power time dependency

**Table 5:** Categorical relationships of 12-month low-back pain (LBP) outcomes to alternative measures of cumulative lifetime vibration dose (quartile based design variables) among professional drivers (N=537, observations=854). See table 2 for the definitions of measures of cumulative lifetime vibration dose. [OR=odds ratio; CI=confidence interval.]

Measures of vibration dose	LBP			Chronic LBP			Sciatic pain		
		
	OR (95% CI) ^a,b^	^2^ test for trend (P-value)	QIC (Δ) ^c^	OR (95% CI) ^a,b^	^2^ test for trend (P-value)	QIC (Δ) ^c^	OR (95% CI) ^a,b^	^2^ test for trend (P-value)	QIC (Δ) ^c^
∑[*a_wz_*^2^*t*] (×10^7 ^m^2^s^-3^)		5.44 (0.020)	742 (0)		4.82 (0.028)	521 (0)		13.0 (0.0003)	989 (0)
0.006–0.57	1.0 (–)			1.0 (–)			1.0 (–)		
0.58–1.34	0.95 (0.50–1.80)			0.90 (0.38–2.14)			0.72 (0.41–1.27)		
1.35–2.70	1.18 (0.62–2.24)			1.06 (0.48–2.35)			1.84 (1.09–3.14)		
2.71–11.7	1.92 (1.05–3.53)			2.25 (1.05–4.81)			2.30 (1.34–3.94)		
∑[*A*(8)*_z_*^2^*t*] (×10^3 ^m^2^s^-4^)		6.52 (0.011)	740 (–2)		5.13 (0.024)	520 (–1)		11.7 (0.0006)	990 (1)
0.003–0.197	1.0 (–)			1.0 (–)			1.0 (–)		
0.198–0.493	0.77 (0.40–1.50)			0.81 (0.34–1.97)			0.75 (0.42–1.32)		
0.494–0.920	1.03 (0.54–1.98)			1.06 (0.48–2.34)			1.69 (0.99–2.89)		
0.921–3.774	2.06 (1.10–3.85)			2.39 (1.08–5.29)			2.30 (1.32–4.02)		
∑[F*_total,rms_*^2^*t*] (×10^11 ^N^2^s)		7.02 (0.008)	738 (–4)		9.84 (0.002)	513 (–8)		14.0 (0.0002)	983 (–6)
0.001–0.17	1.0 (–)			1.0 (–)			1.0 (–)		
0.18–0.45	0.88 (0.46–1.67)			1.03 (0.43–2.50)			1.04 (0.60–1.79)		
0.46–1.01	1.36 (0.72–2.56)			1.56 (0.67–3.61)			1.91 (1.12–3.24)		
1.02–3.57	2.31 (1.17–4.56)			3.81 (1.60–9.10)			2.84 (1.55–5.21)		
∑[F*_total,rms_t*] (×10^9 ^Ns)		7.33 (0.007)	738 (–4)		9.18 (0.0025)	515 (–6)		18.7 (0.0001)	975 (–14)
0.01–0.83	1.0 (–)			1.0 (–)			1.0 (–)		
0.84–1.82	1.23 (0.65–2.35)			1.26 (0.53–2.98)			0.97 (0.56–1.70)		
1.83–3.25	1.32 (0.69–2.52)			1.35 (0.57–3.21)			2.14 (1.26–3.65)		
3.26–8.15	2.69 (1.33–5.42)			4.12 (1.70–10.0)			3.63 (1.94–6.80)		
∑[F*_d,total,peak_* *t*^1/6^] (×10^3 ^N)		5.33 (0.021)	740 (–2)		4.79 (0.029)	521 (0)		11.1 (0.0009)	988 (–1)
0.48–1.37	1.0 (–)			1.0 (–)			1.0 (–)		
1.38–1.94	0.59 (0.30–1.17)			0.45 (0.16–1.25)			0.96 (0.60–1.63)		
1.95–2.55	1.03 (0.55–1.95)			0.94 (0.44–2.02)			1.46 (0.86–2.48)		
2.56–3.96	2.17 (1.10–4.29)			2.46 (1.12–5.43)			2.57 (1.47–4.50)		
∑[Sed*_peak_* *t*^1/6^] (MPa)		3.09 (0.079)	744 (2)		3.33 (0.068)	523 (2)		8.63 (0.0033)	991 (2)
0.26–0.75	1.0 (–)			1.0 (–)			1.0 (–)		
0.76–1.08	0.77 (0.41–1.43)			0.60 (0.24–1.46)			1.05 (0.62–1.78)		
1.09–1.43	1.09 (0.58–2.04)			0.99 (0.46–2.15)			1.29 (0.76–2.19)		
1.44–2.23	1.71 (0.87–3.38)			2.15 (0.95–4.87)			2.47 (1.39–4.36)		

a ∑[*a_wz_*^2^*t*], ∑[*A*(8)*_z_*^2^*t*]: OR adjusted by age-at-entry, body mass index, physical work load, psychosocial work environment, and follow up time.

b ∑[F*_total,rms_*^2^*t*] to ∑[Sed*_peak_* *t*^1/6^]: OR adjusted by age-at-entry, physical work load, psychosocial work environment, and follow up time.

c QIC is the quasi-likelihood information criterion for the comparison between models. QIC difference (Δ) is calculated as the difference between the QIC value for a specific exposure model and the model including ∑[*a_wz_*^2^*t*] calculated according to a second power time dependency.

In previous investigations of the VIBRISKS cohort (11, 12), physical workload, in addition to vibration exposure, was significantly associated with low-back outcomes, while psychosocial work environment, age-at-entry and other individual-related variables (smoking and drinking habits, marital status, annual car driving, education and physical activity) showed no significant relations to LBP outcomes. These findings, however, are beyond the scope of the present study

There were no significant interactions between measures of either acceleration-based or force-based lifetime doses and physical load when appropriate product terms were included in the statistical models.

## Discussion

### LBP outcomes in driving occupations

The findings of this prospective cohort study are in accordance with those of other epidemiological studies and surveys of working populations. A recent systematic literature review and meta-analysis have reported a pooled risk estimate of 2.17 (95% CI 1.61–2.91) for LBP in WBV-exposed worker groups compared to unexposed reference groups, although significant heterogeneity was found between studies (20). Moreover, LBP showed a pooled risk estimate of 1.51 (95% CI 1.30–1.75) by contrasting high- and low-exposure groups of workers, suggesting a possible exposure–response relationship. Similar findings were reported in earlier reviews and meta-analyses (4, 21). In the fifth EWCS 2010 (3), duration of occupational exposures to vibration classified as “often” and “always” of the working time were associated with LBP prevalences of 58.2 and 61.2%, respectively. These figures are consistent with the period prevalence of LBP outcomes (67%) observed in the professional drivers of this study. An excess risk for sciatic pain has been reported in few studies of WBV-exposed workers. In a meta-analysis of nine cross-sectional studies, a pooled risk estimate of 1.92 (95% CI 1.38–2.67) for sciatic pain has been reported in WBV-exposed groups when compared with unexposed reference groups (manual workers, office workers) (20). In this study, a cumulative incidence of 21.8% for sciatic pain was observed in our cohort of professional drivers. This figure is very similar to a three-year cumulative incidence of 22% reported in a longitudinal study of Finnish machine operators who were free from sciatica at baseline (22).

### LBP outcomes and alternative measures of cumulative lifetime vibration dose

The exposure action and limit values for WBV provided by the EU Directive are based on metrics of frequency-weighted acceleration of vibration to which a worker is exposed during a working day, normalized to an 8-hour reference period (5). The protective effectiveness of the EU levels of daily WBV exposure against the risk to the lower lumbar spine and the connected nervous system is not fully known but it is a matter of concern that epidemiological studies have reported low-back troubles among workers exposed to WBV levels lower than the EU exposure action values (8, 12). The metrics *A*(8) and VDV are measures of daily WBV exposure and may be not sufficient to predict the probability of low back disorders which are chronic conditions and can take several years to develop. Furthermore, the current EU WBV metrics for the assessment of vibration related LBP outcomes are measures of external vibration exposure which can reflect only partially the internal dynamic stress on the lumbar spine caused by WBV.

In the present study, the occurrence of LBP outcomes over time was related to a broad variety of external and internal vibration doses by using different exposure quantities (r.m.s. acceleration, r.m.s. force, peak force) integrated over the total lifetime duration of vibration exposure. Vibration exposure expressed in terms of VDV measured by means of the root-mean-quad (r.m.q.) weighting method was not considered in this paper since a previous study of the same cohort of professional drivers revealed that the effects of transient vibration, shocks, or repetitive shocks were better predicted by measures of dynamic compressive stress (eg, Sed) rather than by the VDV metric (11).

Measures of lifetime vibration dose which combine r.m.s. acceleration magnitude with total driving time resulted in improved associations with the occurrence of low-back symptoms compared to those previously observed for the same measures of acceleration magnitude integrated over the daily duration of vibration exposure (11). This is a remarkable finding suggesting that measures of exposure to vibration acceleration accumulated over the work life are more predictive of the development of long-term adverse health effects to the lower back of professional drivers than measures of daily vibration exposure.

Nevertheless, a statistic for model fitting revealed that measures of internal dynamic spinal forces (either r.m.s. force or peak force) combined with total duration of exposure were better predictors of LBP outcomes than acceleration-based lifetime doses. Thus, the findings of this study suggest that metrics of vibration exposure based on cumulative internal spinal load (force) are related to LBP outcomes to a greater extent than those from cumulative external load (acceleration).

In this study, models with force-r.m.s. doses provided a better fit to LBP outcomes than those with force-peak doses. This finding was evident mainly for sciatic pain which is a symptom more severe and with poorer prognosis than low back complaints of non-specific origin. Hence, the differences between the models with force-based doses suggest that the cumulative health effect on the lumbar spine might depend on the integrated resulting total force over the entire time rather than primarily on the force peaks. These findings are consistent with those reported in other epidemiological studies concluding that metrics of cumulative mechanical load are more predictive of the occurrence of LBP outcomes than peak loads (23, 24). This view is also in line with the experimental evidence that vibration and shock are sufficient mechanical risk factors for the initiation and the progression of injuries to the spine tissues supporting a fatigue model for LBP aetiology as the result of accumulation of tissue microdamage and failure to the anatomical structures of the lumbar spine (25, 26).

It may be argued that the method for the measurement of internal spinal forces is too complex to be applicable at a workplace. The force-based method requires several variables to be specified in the input file of the software (measurement/assessment of postures, body mass and height, acceleration time series measured in three directions at the seat surface of the machine(s), daily and lifetime exposure durations) and then processed by means of acceleration to spine-force transfer functions to calculate the spinal forces and the derived metrics for the evaluation of mechanical shocks. In opposite the acceleration-based method requires only the frequency-weighted r.m.s. acceleration (single axis or tri-axial) measured at the machine seat surface and the total duration of exposure to calculate a lifetime acceleration-based dose. Nevertheless, in this study both the acceleration-based doses and the force-based doses were significantly associated with the occurrence of LBP outcomes, although a measure of model fitting showed that the internal force method performed better for the prediction of the adverse health effects on the lumbar spine. These findings suggest that despite the substantial differences in the complexity of the two methods for the evaluation of WBV exposure, they may be considered complementary to each other under certain conditions of exposure. The weighted r.m.s. acceleration-based method, also called the “basic evaluation method” in ISO 2631-1 (6), is suitable for the measurement of periodic, random and transient WBV with crest factor (ie, the ratio of the maximum instantaneous peak value of the frequency-weighted acceleration signal to its r.m.s. value) ≤9. On the other hand, international standard ISO 2631-5 recommends to estimate the internal spinal forces for the evaluation of the biomechanical response of the lumbar spine to vibration containing multiple, repeated, shocks (9). Since the risk assessment methods and related models described in ISO 2631-5 are not yet epidemiologically validated, the findings of this cohort study tend to support the force-based method for assessing the risk of health impairment to the lumbar spine due to long-term exposure to WBV containing multiple mechanical shocks.

### Limitations of the study

There are limitations and uncertainties in this study that deserve attention (11, 12). Some limitations are linked to the exposure data. Since vibration measurements were made on currently available machines or vehicles, uncertainty in the estimation of lifetime vibration doses may arise. Nevertheless, the weighted r.m.s. acceleration magnitude of vibration measured in the vehicles of the present study are comparable with those reported in recent and past investigations (4, 27–29). In this study, the acceleration-based doses were constructed using only the vibration acceleration weighted in the *z*-direction (vertical). As aforementioned, this was done for purposes of comparison with the metrics for vibration shocks recommended by ISO 2631-5 (9). It should be noted, however, that the *z*-axis (vertical) weighted acceleration (*a_wz_*) was the dominant directional component of vibration measured in all of the machines and vehicles (7, 12). Considering the multiplying factor 1.4 for the horizontal axes according to EU Directive on mechanical vibration (5), only one vehicle during a special activity (wheel loader, stone transportation in a quarry) revealed a dominant directional component in the *x*-axis (7).

Estimation of daily and weekly driving times with exposure to WBV by means of questionnaire methods may be subject to recall bias. In this study additional information was retrieved from company records and direct interviews to employees and employers. The daily exposure patterns were assumed to be constant over the working life. Some subjects operated one machine exclusively; other subjects operated a combination of two or three vehicles. If two or three vehicles were driven per day, an equal proportion of time per day was assumed (one-half and one-third, respectively), and the time histories of the spinal forces in response to each vehicle were combined to calculate the overall internal spinal load.

The questionnaire used in this study was originally developed within the European project VINET (14). The questionnaire underwent a process of improving revisions on the basis of the findings of pilot studies and epidemiological surveys conducted across some European countries (14). Although the role of the questionnaire as an instrument to collect exposure data is still controversial (30), questionnaire methods may offer a means for studying cumulative exposure over time, a variable which cannot be estimated by direct observations or measurements (31).

In this study the spinal response to WBV has been investigated by means of FE-modelling based on the real anatomy of the lumbar spine region. The FE-models have been validated by means of experimental laboratory research on human biodynamics and field research on anthropometry and posture of representative groups of European drivers (10, 32–34). Present limitations of the FE-models concern its linearity, simplified modelling of soft tissue at the input areas, missing consideration of twisting, and limited verification for horizontal and rotational inputs (7). The current FE-models to calculate spinal forces during exposure to WBV do not provide a quantitative risk assessment for internal stresses caused by shear forces, bending and torsion, since reliable strength data (ultimate static strength, strength under dynamic repetitive load) for such stresses are not available.

### Concluding remarks

In this prospective cohort study, metrics of cumulative vibration exposure based on either external (acceleration-based) or internal (force-based) spinal load were significantly associated with the occurrence of LBP outcomes in professional drivers exposed to WBV containing multiple mechanical shocks. Measures of internal lumbar forces were better predictors of LBP outcomes than measures of external vibration acceleration although the exposure metrics constructed with the acceleration-based method have the advantage of greater simplicity compared to the force-based method. Models with force-r.m.s. doses provided a better fit to LBP outcomes than those with force-peak doses. These findings suggest that the cumulative health effect on the lumbar spine might depend on the integrated resulting total force over the entire time rather than primarily on the force peaks.
